# A Comparative Study of the Epidemiology of Treponemal Infection in the Volta and Oti Regions of Ghana: A Five-Year Multisite Parallel Population-Based Analysis vis-à-vis the Sentinel Survey

**DOI:** 10.1155/2021/4462389

**Published:** 2021-11-09

**Authors:** Sylvester Yao Lokpo, Ellis Owusu-Dabo, John Gameli Deku, Verner Ndudiri Orish, Gideon Kye-Duodu, Francis Abeku Ussher, Thomas Boakye, Daniel Adigbli, Louis Selassie Ameke, William Klutse Fianko, Robert Adedze-Kpodo, Henry Komla Letsa, Worlanyo Tashie, Noble Selorm Gbormittah, Godsway Edem Kpene, James Osei-Yeboah

**Affiliations:** ^1^Department of Medical Laboratory Sciences, School of Allied Health Sciences, University of Health and Allied Sciences, Ho, Ghana; ^2^School of Public Health, Kwame Nkrumah University of Science and Technology, Kumasi, Ghana; ^3^Department of Microbiology and Immunology, School of Medicine, University of Health and Allied Sciences, Ho, Ghana; ^4^Department of Epidemiology and Biostatistics, School of Public Health, University of Health and Allied Sciences, Ho, Ghana; ^5^Faculty of Health and Allied Sciences, Koforidua Technical University, Koforidua, Ghana; ^6^Laboratory Department, Krachi West District Hospital, Krachi, Ghana; ^7^Laboratory Department, Ho Municipal Hospital, Ho, Ghana; ^8^Laboratory Department, Hohoe Municipal Hospital, Hohoe, Ghana; ^9^Laboratory Department, District Hospital, Sogakope, Ghana

## Abstract

Treponemal infections can be blood-borne with great public health consequences. This study is aimed at comparatively describing the five-year (2013-2017) regional epidemiology of treponemal infection using pregnant women in the sentinel survey and apparently healthy blood donors as a proxy for the general population at four sentinel sites in the Volta and Oti Regions of Ghana. We analyzed retrospective data from 17,744 prospective blood donors aged 18 to 58 years and 7,817 pregnant women in a sentinel survey with ages from 15 to 49 years at Hohoe, Ho, Tongu, and Krachi West sentinel sites in the Volta and Oti Regions. Laboratory data extracted include variables such as age, gender, date of blood donation, and *Treponema pallidum* chromatographic immunoassay results from the blood banks of the four study sites. The five-year treponemal infection rate among the pregnant women in the sentinel survey and prospective blood donors was 0.79% and 2.38%, respectively. Site-specific infection rate for population-based/sentinel survey was 4.6%/1.1%, 2.0%/0.5%, 1.3%/1.1, and 1.2%/0.3% for Hohoe, Ho, Krachi West, and Tongu, respectively. Significant gender disparity in Treponemal infection rate exists with a male preponderance. The regional infection rate in the sentinel survey is lower compared to the general population. Therefore, the use of pregnant women as a proxy for population estimates could underestimate the burden in the study jurisdiction.

## 1. Introduction

Treponematoses are bacterial diseases caused by *Treponema pallidum* subspecies *pallidum* and *pertenue.* They are a major public health problem worldwide [1]. Treponematosis exists mainly in two forms: venereal and nonvenereal disease. Nonvenereal treponematoses include yaws, bejel, and pinta; these are not sexually transmitted and thus are not important if found in the blood supply. However, syphilis, an example of venereal treponematosis can be acquired via unprotected sexual intercourse with an infected person [2]. According to statistics released in 2019 by the World Health Organization (WHO), one percent or more of antenatal care attendees in 38 of 78 reporting countries tested positive for syphilis [3] while among blood donors an estimated 1.6 million units of blood were discarded due to the presence of infectious markers including those caused by Treponemal species [4]. In sub-Saharan Africa, syphilis infection in women of reproductive age group is reported to range from 0.36% to 3.6% [5] whereas rates ranging from 0.71% to 20% have been recorded among blood donors [6, 7]. In Ghana, a nationally representative survey in 2017 revealed that syphilis infection prevalence among pregnant women, as well as healthy blood donors, varied between 0.3% and 3.7% [8, 9]. Mother-to-child transmission may lead to adverse pregnancy outcomes, including foetal loss, neonatal death, low birth weight, stillbirth, and congenital abnormalities [10]. It was estimated that over 350 000 adverse birth outcomes including 200 000 stillbirths and newborn deaths were recorded worldwide in 2016 [11]. However, some people can be infected without showing signs and symptoms thus becoming carriers for a long period which in turn might lead to the spread of the infection [12].

There have been concerns about whether estimates from sentinel surveys of pregnant women could represent the infection rates in the general population. This called for the recommendation that periodic validations of antenatal clinic (ANC) surveillance data are carried out through the conduct of population-based studies in countries with generalized epidemiology [13]. Pursuant to this, we previously described the epidemiology of syphilis infection by comparing estimates from the sentinel survey with those observed among blood donors as a proxy population at the Ho sentinel site in the Volta Region [14]. We found that the use of pregnant women might underestimate the population burden of the infection. Moreover, understanding the epidemiological pattern of treponemal infections from a regional perspective could be an important step to reviewing national strategies aimed at eliminating the menace from the Ghanaian population. Hence, using a similar approach but a widened scope, we aimed to describe the five-year (2013-2017) regional epidemiology of treponemal infection by comparing estimates from the sentinel survey with blood donors as proxy for the asymptomatic adult population at four sentinel sites in the Volta and Oti Regions of Ghana.

## 2. Materials and Methods

### 2.1. Study Area and Study Sites

The Volta and Oti Regions are part of the sixteen (16) administrative regions located in the eastern part of Ghana. It is bounded by the Northern Region to the north, south by the Gulf of Guinea, west by the Volta Lake, and east by the Republic of Togo. The two regions occupy a total surface area of about 20,570 square kilometers. The population of the two regions based on the national population and housing census in 2010 was 1,901,179 with an annual growth rate of 1.9%. The largest populated district is Ho, with a population of 214,612 followed by Hohoe municipal with a population of 181,297. The least populated district is Jasikan with a population of 58,483.

### 2.2. Study Design and Study Population

This study is a retrospective analysis of secondary data from 17,744 prospective blood donors aged from 18 to 58 years and 7,817 pregnant women in a Sentinel Survey who were within 15- and 49-year age bracket. The study was conducted at four HIV/STI sentinel sites in the Volta and Oti Regions, namely, Ho, Hohoe, Krachi West, and Tongu. Ho and Hohoe sites are located in urban Ghana, while Krachi West and Tongu sites are of rural settings [15].

### 2.3. Data Collection

Data on submitted samples (including test results) of treponemal infection for pregnant women at the four sentinel sites in the Volta and Oti Regions were extracted from the laboratory records spanning the period 2013-2017. Laboratory data on treponemal test results from the blood banks of the four study sites were also extracted. In both study populations, data on demographic variables including age, gender, and date of donation were obtained. All four sites employed chromatographic immunoassays for the qualitative detection of antibodies to *Treponema palladium* in human serum using the First Response test kits (Premier Medical Corporation Private Limited, India). The sensitivity and specificity of the assay were 100% and 99.18%, respectively, after external evaluation. In brief, two purified recombinant *TP* antigens are made into a test capture band material and a gold conjugate. The presence of *TP* antibodies in a serum test sample forms a complex with the immobilized antigens in the test zone of the membrane leading to a visible pink-rose coloured band on the membrane.

### 2.4. Data Analysis

Data were collected, entered into Microsoft Excel 2016 spreadsheet, and validated for entry errors. Data were presented as frequencies and proportions. Differences between proportions and trends analyses were carried out using Fisher's exact test, and chi-square test for trend where appropriate. A *p* value < 0.05 was considered statistically significant. IBM Statistical Package for the Social Sciences (SPSS Inc. Chicago, USA; http://www.spss.com/) version 22.0 was used for analysis.

### 2.5. Ethical Considerations

Approval for this study was obtained from the authorities of the four sentinel hospitals. Ethical clearance for the study was obtained from the Research Ethics Committee of the University of Health and Allied Sciences, Ho (UHAS-REC A.4 [171] 18-19). The data retrieved were anonymous and nonlinked. Patients' names and other attributes that could lead to identity disclosure were not retrieved from the archives.

## 3. Results

The five-year prevalence of treponemal infections in the Volta and Oti Regions was estimated as 0.79% by the sentinel survey; 2.38% infection rate was observed in the population-based survey using all prospective blood donors and 2.31% infection rate among prospective donors within the sentinel survey age range (15-49 years). The year-on-year regional epidemiological pattern in the sentinel survey in general recorded a decreasing trend (p for trends 0.0025), moving to under 1% rate in the last three years of the period under review. Except for 2017, the estimated regional treponemal infection in the population-based sample was above 2% in each of the review years. Statistically higher population estimates were observed for years 2013, 2015, and 2016. An undulating trend was observed for the population-based estimates, peaking in 2016, and troughing in the following year (2017) Table 1.

In the multisite analysis, significant site-specific differences in the cumulative treponemal infection rate were observed (*p* < 0.0001). Hohoe with a prevalence of 4.6% recorded the highest burden of treponemal infection within the five years of the review. Ho sentinel site was second (2.02%) followed by Krachi West (1.34%) and Tongu (1.18%). Except for Tongu, which recorded the highest treponemal infection in the year 2016 (2.08%), the peak year of treponemal infection was observed in the year 2013 for all sites. The epidemiology pattern of treponemal infection, in general, observed a decline at the end of the review year (2017) from the previous year (2016) in the four sites. However, the margin of reduction in the case of Tongu was found to be statistically not significant (*p* = 0.5016).  Table 2.

**Figure 1:**
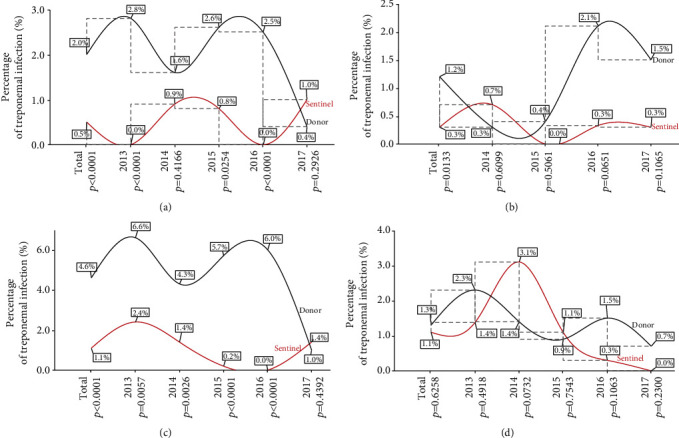
Year-on-year site specific treponemal prevalence parallel analysis with the sentinel survey at the four Volta Region sentinel sites. (a) Ho sentinel site, (b) Tongu sentinel site, (c) Hohoe sentinel site, and (d) Krachi West sentinel site. *p* is significant at 0.05.

The five-year rate of treponemal infection was found to be significantly higher in the population-based sample compared to the sentinel survey in all sentinel sites except Krachi West. The infection pattern in both populations at all sentinel sites presented an undulating trend in the year-on-year treponemal infection rate. Though not statistically significant in all cases, the population-based study presented a higher year-on-year treponemal infection rate. However, the 2014 rate in Tongu and Krachi West, the 2015 rate in Krachi West, and the 2017 rates in both Ho and Hohoe sites bucked the trend with higher rates in the sentinel survey. Except for Tongu which as of 2013 was not a sentinel site, the peak year in the population-based sample for all the other sites was at the beginning of the review period (2013) and troughed at the end of the review period (2017) (Figure 1).

**Figure 2:**
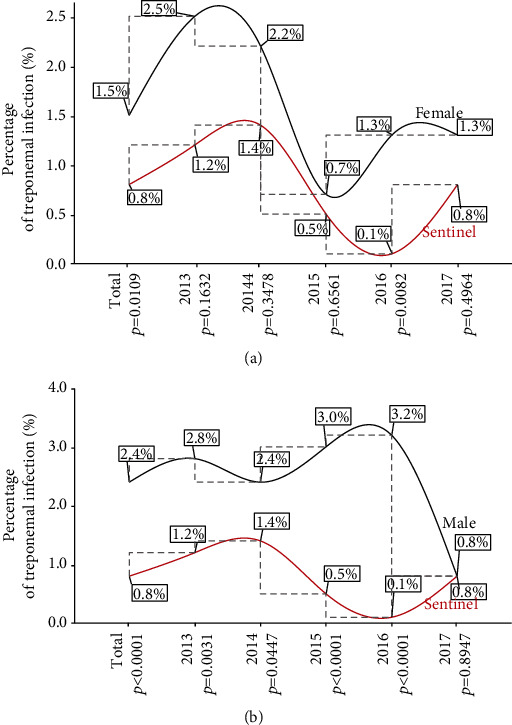
Year-on-year treponemal prevalence parallel analysis with the sentinel survey at the four Volta Region sentinel sites. (a) Female and (b) male. *p* is significant at 0.05.

The five-year prevalence of treponemal infection was observed at a 1.5% rate among the female population (Figure 2(a)) and 2.4% rate among their male counterparts, while the sentinel estimate was 0.8% (Figure 2(b)). There is a significant male preponderance in the five-year infection rate. As seen in Figure 2(a), estimates among the females were found to be higher in each of the years under review, though the statistically significant difference was observed only at one point in time (2016). Except for 2017, the year-on-year review revealed significantly higher rates among the male population compared to estimates from the sentinel survey (Figure 2(b)).

## 4. Discussion

To the best of our knowledge, this is the first comparative study aimed at describing the epidemiological pattern of treponemal infection within a regional context taking into consideration four HIV/STI sentinel sites (Ho, Hohoe, Tongu, and Krachi West) in the Volta and Oti Regions. The rate of treponemal infection over the five-year review period (2013-2017) was 0.79% among 7,817 pregnant women in the sentinel survey and 2.38% in the population-based survey among 17,744 apparently healthy blood donor population. Moreover, the five-year infection rate observed among the asymptomatic adult population (16, 858) who fell within the sentinel age group (15-49 years) was 2.31% (Table 1). The infection rates recorded in this study are lower compared to the 5.1% reported in Uganda [16] and 7.2% in Tanzania [17]. However, similar results have been published previously by us [18] and in studies from other countries including 0.5% in South Africa [19] and 2.9% in Hungary [20]. Although this study recorded a relatively low infection rate in comparison to other settings, the findings suggest that treponemal infection is a public health problem in the Volta and Oti Regions. Hence, this could pose a challenge to efforts by the WHO to achieve total elimination of congenital transmission of treponemal infection [21].

In general, the five-year and yearly treponemal infection rates were significantly higher in the population-based group compared to those recorded in the sentinel survey, except for the year 2017 where the difference was comparable between the two groups [sentinel survey vs. general population: 13 (0.78) vs. 33 (0.82); *p* = 0.9960] (Table 1). Similarly, a comparison of the infection rates between the female donor population and data from the sentinel survey revealed significantly higher rates in the cumulative five years and year 2016 among the donor population (Figure 2(a)). Thus, our earlier finding of a higher rate of treponemal infection among the asymptomatic adult population compared to the sentinel survey is not limited to the Ho sentinel site [14] but represents a regional picture. Previous reports in other African countries also corroborated the results of this study. Studies in Tanzania [22–24], Uganda [25], and Zambia [26] reported a 10-30% lower prevalence of sexually transmitted infections in pregnant women compared to individuals in the general population. Thus, the finding of a lower rate of treponemal infection in the sentinel survey compared to the population-based group raises important question as to whether estimates from sentinel surveys can accurately represent those in the general population within the Volta and Oti Regions. Boisson et al. [27] and Zaba and Gregson [28] have posited that biases exist for using pregnant women as a proxy estimate of sexually transmitted infections in the general population. In the view of Gregson et al. [29], not all pregnant women would seek antenatal services at a health facility while Gonese et al. [30] argued that the use of pregnant women for population estimates would exclude nonpregnant women of similar reproductive age group as well as men in the general population.

In the multisite analysis, we found that the five-year treponemal infection rate was highest in Hohoe (4.6%), followed by Ho (2.02%), Krachi West (1.34%), and Tongu (1.18%) among blood donors (Table 2) while a rate of 1.1% was recorded in both Hohoe and Krachi West and 0.5% and 0.3% for Ho and Tongu sentinel sites, respectively, among pregnant women during the five years under review. The five-year prevalence was significantly higher in the population-based sample compared to the sentinel survey at all sentinel sites except Krachi West, while the yearly infection pattern in both populations in all the sentinel sites presented with a characteristic undulating trend (Figures 1(a)–1(d)). Hohoe and Ho sentinel sites are located within urban Ghana while Krachi West and Tongu sites are of rural origin [15]. There is a strong and consistent link between conditions prevalent in urban areas and the spread of sexually transmitted infections. Factors including urban poverty due to socioeconomic inequalities could lead to the dependence of girls and women on men who are gainfully employed for economic survival and the resultant increased rate of unintended pregnancies and sexually transmitted infections [31].

In the present study, we observed a significant gender disparity of treponemal infection for the five years under review. The male subpopulation of blood donors recorded higher infection rates compared to pregnant women in the sentinel survey in the cumulative five-year and the yearly infection rate from 2013-2016, except for 2017 where the rates were statistically comparable (Figure 2(b)). The results suggest a higher risk of infection among male donors compared to pregnant women. The finding is consistent with our previous result in Ho [14] and the works of Anwar et al. [32]. However, our finding contradicts the reports of Coffin et al. [33] and Gao et al. [34] where females were more susceptible to treponemal infection compared to their male counterparts.

This study has a limitation worth mentioning, hence, any interpretation should be made in the light of this limitation. The diagnostic method adopted in screening participants (chromatographic immunoassay to qualitatively detect antibodies to *Treponema palladium* (*TP*) in human serum) limits the ability to distinguish between active cases and past or treated infections. However, the strength of this paper lies in the scope of coverage (four sentinel sites) which gives a regional perspective of the rate of treponemal infection.

## 5. Conclusion

The regional prevalence of treponemal infection in the Sentinel Survey is lower compared to the general population. Therefore, the use of pregnant women as proxy for the population estimates could underestimate the infection rate in the Volta and Oti Regions of Ghana.

## Figures and Tables

**Table 1:** Year-on-year prevalence of treponemal infection among blood donors and pregnant women (sentinel survey) in the Volta Region.

Parameter	Total	2013	2014	2015	2016	2017	*p* for trends
*Sentinel*	7817	1292	1525	1720	1605	1675	
Positive	62 (0.79)	16 (1.24)	22 (1.44)	9 (0.52)	2 (0.12)	13 (0.78)	0.0025
*All donors*	17744	2753	3195	3336	4421	4039	
Positive	423 (2.38)	78 (2.83)	75 (2.35)	93 (2.79)	144 (3.26)	33 (0.82)	0.0001
*Donors (within the sentinel age group)*	16858	2550	3036	3160	4250	3862	
Positive	389 (2.31)	69 (2.71)	70 (2.31)	86 (2.72)	131 (3.08)	33 (0.85)	0.0002
*p*	<0.0001	<0.0025	0.0525	<0.0001	<0.0001	0.9960	
*p*1	<0.0001	<0.0050	0.0651	<0.0001	<0.0001	0.8935	

Data are presented as the frequency with the corresponding percentage in parenthesis. *p* compares all donors within the sentinel, and *p*1 compares donor within 15-49 years with the sentinel. *P* is significant at 0.05.

**Table 2:** Site-specific year-on-year distribution of treponemal infection among blood donors.

Parameter	Total	2013	2014	2015	2016	2017	*p* for trends
Ho	85/4212 (2.02)	32/1126 (2.84)	10/630 (1.59)	17/646 (2.63)	22/892 (2.47)	4/918 (0.44)	0.0031
Tongu	25/2127 (1.18)	2/225 (0.89)	1/300 (0.33)	2/464 (0.43)	11/529 (2.08)	9/609 (1.48)	0.0610
Hohoe	226/4908 (4.60)	18/273 (6.59)	48/1121 (4.28)	64/1122 (5.70)	87/1449 (6.00)	9/943 (0.95)	0.0013
Krachi West	87/6497 (1.34)	26/1129 (2.30)	16/1144 (1.40)	10/1104 (0.91)	24/1551 (1.55)	11/1569 (0.70)	0.0030
*p* value	<0.0001	0.0004	<0.0001	<0.0001	<0.0001	0.1442	

Data are presented as frequency (positive/total) with the corresponding percentage in parenthesis. *p* is significant at 0.05. *p* for trends: evaluating significant yearly linear trends; *p*: an intersite comparison.

## Data Availability

The datasets used during the current study are available from the corresponding author on request.
